# Development of an ICF‐Based Core Assessment Set for Psychiatric Occupational Therapy in Japan: A Three‐Round Delphi Study

**DOI:** 10.1155/oti/5138252

**Published:** 2026-05-04

**Authors:** Kojiro Kawano, Shigehito Shiota, Kotomi Funatsu, Chiga Murai, Takashi Tamura, Takaji Suzuki, Toshio Higashi, Shinichi Noto

**Affiliations:** ^1^ Japanese Association of Occupational Therapists, Taito, Japan; ^2^ Department of Rehabilitation, Graduate School of Health Sciences, Saitama Prefectural University, Saitama, Japan, spu.ac.jp; ^3^ Department of Rehabilitation, Hiroshima University Hospital, Hiroshima, Japan, hiroshima-u.ac.jp; ^4^ Medical Corporation Yuaikai, Tikumaso Mental Hospital, Nagano, Japan; ^5^ Ishikawa Prefectural Hospital of Mental Health, Kahoku City, Ishikawa, Japan; ^6^ Akita Rosai Hospital, Akita, Japan; ^7^ Graduate School of Biomedical Sciences, Nagasaki University, Nagasaki, Japan, nagasaki-u.ac.jp; ^8^ Department of Rehabilitation, Niigata University of Health and Welfare, Niigata, Japan, nuhw.ac.jp; ^9^ Department of Occupational Therapy, Kinjo University, Nagoya, Ishikawa, Japan

**Keywords:** assessment tool, Delphi method, ICF, International Classification of Functioning Disability and Health, mental disorders, occupational therapy

## Abstract

**Introduction:**

People with mental disorders have an increasing need for a comprehensive assessment tool based on the International Classification of Functioning, Disability, and Health (ICF). This study is aimed at identifying ICF categories relevant to patients with mental health in Japan using a Delphi panel of experts and provide foundational data for developing an occupational‐therapy–oriented assessment tool.

**Methods:**

A three‐round Delphi survey was conducted with a Japanese expert panel initially composed of 15 specialists (five psychiatrists, 10 occupational therapists). Questionnaires (mailed or web‐based) were developed from the ICF Checklist, existing ICF core sets, relevant clinical guidelines, and prior research. Aggregate results were submitted to participants after each round for re‐evaluation. Fourteen experts completed all rounds. Data were collected from August 2024 to January 2025. Consensus was determined according to the RAND/UCLA appropriateness method.

**Results:**

Based on consensus, 42 ICF categories were selected: 17 body functions, 21 activities and participation, and four environmental factors. The set included a relatively large proportion of activities/participation items, reflecting the emphasis of occupational therapy on engagement and participation. Agreement rates increased across rounds, indicating improving consensus and relative stability of the selected indicators.

**Conclusion:**

This Delphi study produced a preliminary ICF set applicable to patients with mental health in Japan and offers the first step toward an ICF‐based comprehensive assessment tool for occupational therapy. Future work should evaluate the reliability and validity of the extracted core set, assess its clinical utility, and incorporate perspectives of service users and families.

## 1. Introduction

The global burden of mental disorders is rising, and the number of people living with mental illness in Japan was approximately 6.03 million in 2023 [1]. Various factors have been found to worsen psychiatric symptoms and outcomes—including symptom severity, relapse episodes, medication nonadherence, presence of triggers, overall functioning, lack of social contact and employment, and negative life events [2–4]. These findings highlight the need to assess not only diagnostic data but also functioning, disability, environmental, and personal factors using a comprehensive framework such as the International Classification of Functioning, Disability, and Health (ICF).

The ICF, published by the World Health Organization in 2001, provides a biopsychosocial framework that classifies human functioning aspects into approximately 1500 codes [5]. Occupational therapists commonly use the ICF to gain a holistic understanding of clients′ lives and guide intervention, education, and support. However, the large number of ICF codes creates practical barriers to routine clinical use. To address this complexity, the World Health Organization developed ICF core sets, defined as concise, evidence‐based lists of ICF categories that reflect the typical spectrum of problems experienced by people with specific health conditions [6]. Core sets are derived from literature reviews, expert input, and empirical data, and several ICF core sets have been developed for psychiatric conditions, such as schizophrenia, bipolar disorder, and depression [7–9]. The core sets have been translated into Japanese and appear in related publications [10, 11]. Given that healthcare systems, social services, and cultural contexts differ across countries, tools developed elsewhere may not align fully with the needs and practices of Japanese clinical settings. Therefore, an ICF‐based assessment instrument tailored to Japan′s cultural, institutional, and occupational‐therapy contexts—hereafter referred to as a Core Assessment Set (CAS)—need to be developed.

In psychiatric occupational therapy, assessment has traditionally emphasized symptoms and diagnostic categories, with less systematic incorporation of functioning and participation perspectives [10, 12]. Consequently, comprehensive interventions aimed at recovery and improved quality of life have sometimes been difficult to plan and implement in a systematic way [10]. Existing assessment scales are often disease specific or focus on limited functional domains, leaving clinicians—particularly occupational therapists—uncertain about the priority domains [13]. A CAS that organizes body functions, activities and participation, and environmental factors within a standard international framework could improve the transparency and reproducibility of assessment, facilitate a common language across professions, and serve as an educational tool for clinicians, students, and trainees.

This study was designed to collect expert consensus on ICF categories relevant to people with mental disorders in Japan, with the aim of producing a preliminary CAS for psychiatric occupational therapy. By selecting items that reflect Japan′s cultural and service contexts and by emphasizing activity and participation domains, this research provides the first step toward a context‐sensitive, standardized assessment framework that supports both clinical decision‐making and cross‐national research comparisons.

## 2. Materials and Methods

### 2.1. Study Design

**Figure 1:**
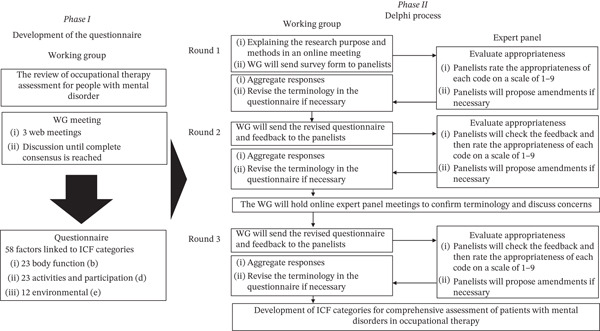
Development of the questionnaire and Delphi process. Phase 1 presents the steps taken by the working group to develop the questionnaire, whereas Phase 2 presents the Delphi survey procedure carried out with the expert panel.

We conducted a prospective, three‐round Delphi survey between August 2024 and January 2025 to select ICF categories for a comprehensive assessment of people with mental disorders. In this study, we adopted a consensus exercise rather than other approaches because it was necessary to ensure a high level of both content validity and consistency with clinical practice in the selection of ICF categories. A multidisciplinary working group (WG)—including all authors—developed the questionnaire based on a literature review. The questionnaire asked panelists to rate the appropriateness of each ICF category for a comprehensive assessment of people with mental disorders using a 9‐point Likert scale ranging from 1 (*not at all appropriate*) to 9 (*very appropriate*). All questions included free‐text boxes for optional comments. Candidate items were extracted from the ICF Checklist [14], existing ICF core sets for psychiatric conditions [7–9], relevant clinical practice guidelines [15–19], and a preliminary survey [13]. The WG members, who are occupational therapists with extensive clinical and rehabilitation experience in mental health, discussed ICF categories specific to psychiatric practice and iteratively refined questionnaire items through face‐to‐face workshops and email exchanges. The study materials and questionnaire were not pilot‐tested prior to the Delphi survey. The ICF items and their sources are listed in Table 1, and this source information (evidence basis) was also provided to the panelists within the questionnaire. Following standard practice for ICF core set development, the Delphi procedure comprised three rounds of anonymous, controlled‐feedback questionnaires administered by mail or via web, with all responses anonymized. Items that met the prespecified consensus criteria were retained and included in the subsequent voting rounds. Psychiatrists received an honorarium for each completed round of the survey. Each round lasted approximately 4 weeks to complete. Initially, participants were given 2 weeks to respond, followed by a reminder and, if necessary, an extension of up to two additional weeks. A flowchart of the study process is presented in Figure 1. The study procedures adhered to the key features of Delphi method, including anonymity, iterative controlled feedback, statistical group response, and solicitation of expert opinion [20, 21]. This consensus study was conducted with support from the Japanese Association of Occupational Therapists (JAOT). JAOT assisted with recruiting the expert panel, arranging web meetings, mailing the questionnaires, and paying honoraria to psychiatrists. The study protocol was not prospectively registered on any public trial registration platform.

**Table 1:** Selection rationale and sources of ICF codes.

No.	ICF code	Evidence category
1	b110—Consciousness functions	ICF Checklist
2	b114—Orientation functions	ICF Checklist
3	b117—Intellectual functions	ICF Checklist and preliminary survey/review
4	b122—Global psychosocial functions	ICF core set (schizophrenia)
5	b126—Temperament and personality functions	ICF core set (bipolar disorders and depression) and preliminary survey/review
6	b130—Energy and drive functions	ICF Checklist, ICF core set (schizophrenia, bipolar disorders, and depression), and preliminary survey/review
7	b134—Sleep functions	ICF Checklist, ICF core set (bipolar disorders), and preliminary survey/review
8	b140—Attention functions	ICF Checklist, ICF core set (schizophrenia, bipolar disorders, and depression), and preliminary survey/review
9	b144—Memory functions	ICF Checklist and ICF core set (bipolar disorders)
10	b147—Psychomotor functions	ICF core set (depression)
11	b152—Emotional functions	ICF Checklist, ICF core set (schizophrenia, bipolar disorders, and depression), and preliminary survey/review
12	b156—Perceptual functions	ICF Checklist, ICF core set (schizophrenia), and preliminary survey/review
13	b160—Thought functions	ICF core set (schizophrenia, bipolar disorders, and depression) and preliminary survey/review
14	b164—Higher level cognitive functions	ICF Checklist, ICF core set (schizophrenia), and preliminary survey/review
15	b167—Mental functions of language	ICF Checklist and preliminary survey/review
16	b180—Experience of self and time functions	ICF core set (schizophrenia)
17	b420—Blood pressure functions	ICF Checklist
18	b455—Exercise tolerance functions	Clinical practice guideline (CPG)—disorder specific, pharmacotherapy, and occupational therapy
19	b515—Digestive functions	ICF Checklist
20	b530—Weight maintenance functions	ICF Checklist
21	b540—General metabolic functions	Clinical practice guideline (CPG)—disorder specific, pharmacotherapy, and comorbidity prevention/management
22	b545—Water, mineral, and electrolyte balance functions	Clinical practice guideline (CPG)—disorder specific and pharmacotherapy
23	b730—Muscle power functions	ICF Checklist
24	d155—Acquiring skills	ICF core set (schizophrenia)
25	d163—Thinking	ICF core set (depression)
26	d175—Solving problems	ICF Checklist and ICF core set (schizophrenia, bipolar disorders, and depression)
27	d177—Making decisions	ICF core set (depression)
28	d210—Undertaking a single task	ICF Checklist and preliminary survey/review
29	d220—Undertaking multiple tasks	ICF Checklist and preliminary survey/review
30	d230—Carrying out daily routine	ICF core set (schizophrenia, bipolar disorders, and depression) and preliminary survey/review
31	d240—Handling stress and other psychological demands	ICF core set (schizophrenia, bipolar disorders, and depression) and preliminary survey/review
32	d350—Conversation	ICF Checklist, ICF core set (depression), and preliminary survey/review
33	d465—Moving around using equipment	ICF Checklist
34	d510—Washing oneself	ICF Checklist, ICF core set (depression), and preliminary survey/review
35	d570—Looking after one′s health	ICF Checklist and ICF core set (schizophrenia, bipolar disorders, and depression)
36	d620—Acquisition of goods and services	ICF Checklist
37	d630—Preparing meals	ICF Checklist
38	d640—Doing housework	ICF Checklist and preliminary survey/review
39	d710—Basic interpersonal interactions	ICF Checklist, ICF core set (schizophrenia), and preliminary survey/review
40	d720—Complex interpersonal interactions	ICF Checklist and ICF core set (schizophrenia)
41	d740—Formal relationships	ICF Checklist
42	d760—Family relationships	ICF Checklist, ICF core set (schizophrenia, bipolar disorders, and depression)
43	d770—Intimate relationships	ICF Checklist and ICF core set (bipolar disorders and depression)
44	d845—Acquiring, keeping, and terminating a job	ICF core set (schizophrenia, bipolar disorders, and depression) and preliminary survey/review
45	d910—Community life	ICF Checklist and ICF core set (schizophrenia)
46	d920—Recreation and leisure	ICF Checklist and preliminary survey/review
47	e1101—Drugs	ICF Checklist and ICF core set (bipolar disorders and depression)
48	e310—Immediate family	ICF Checklist and ICF core set (schizophrenia and depression)
49	e320—Friends	ICF Checklist and ICF core set (bipolar disorders and depression)
50	e325—Acquaintances, peers, colleagues, neighbors, and community members	ICF Checklist and ICF core set (depression)
51	e355—Health professionals	ICF Checklist and ICF core set (schizophrenia, bipolar disorders, and depression)
52	e410—Individual attitudes of immediate family members	ICF Checklist and ICF core set (schizophrenia, bipolar disorders, and depression)
53	e415—Individual attitudes of extended family members	ICF core set (depression)
54	e420—Individual attitudes of friends	ICF Checklist and ICF core set (depression)
55	e450—Individual attitudes of health professionals	ICF Checklist and ICF core set (schizophrenia and depression)
56	e460—Societal attitudes	ICF Checklist and ICF core set (schizophrenia and bipolar disorders)
57	e570—Social security services, systems, and policies	ICF Checklist and ICF core set (schizophrenia)
58	e580—Health services, systems, and policies	ICF Checklist and ICF core set (schizophrenia and depression)

### 2.2. Expert Panel

The expert panel initially comprised 15 specialists: five psychiatrists and 10 occupational therapists. Psychiatrists were nominated by professional organizations, including the Japanese Society of Psychiatry and Neurology, the Japanese Association of Psychiatric Hospitals, and the National Association of Public Hospitals. All psychiatrist panelists were designated psychiatrists with experience in occupational therapy and had participated in committees organized by JAOT. Occupational therapists were recommended by JAOT and were selected on the basis of their prior participation in specialist training sponsored by public psychiatric hospital associations and experience in case reporting using the ICF. Occupational therapists had a mean (SD) of 21.1 (8.61) years of professional experience, and psychiatrists had 40.4 (5.08) years.

### 2.3. Data Collection and Analysis

In Round 1, the WG distributed the questionnaires, together with background information and instructions, to the 15 panelists. The panelists were asked to indicate the ICF categories they considered necessary for a comprehensive assessment of people with mental disorders. The WG calculated selection rates for each category and reviewed the results in a web meeting. Based on these discussions, some items were removed or added, and the Round 2 questionnaire was prepared.

Round 2 questionnaires, together with the aggregated Round 1 results, were sent to participants. Again, the panelists selected ICF categories via Google Forms or by return mail. Selection rates were recalculated and discussed in a second web meeting. The WG further revised the item list, creating the Round 3 questionnaire.

In Round 3, the Round 2 results and the final questionnaire were distributed. The Round 3 responses were used to determine the ICF categories deemed necessary for clinical intervention. Thus, the WG revised the questionnaire between rounds and completed three iterative surveys.

Collected data were tabulated and categorized into three levels of appropriateness using the criteria described in the RAND/UCLA Appropriateness Method User′s Manual [22]:

Appropriate: panel median 7–9 with no disagreement.

Uncertain: panel median 4–6 or median in the disagreement range.

Inappropriate: panel median 1–3 with no disagreement.

Between rounds, the WG reviewed the panel ratings together with free‐text comments and used these materials to refine the questionnaire for the subsequent round. Specifically, the WG organized panel suggestions, removed redundant or overlapping candidate items, and considered whether additional categories proposed by panelists should be included in the next‐round questionnaire. Thus, WG judgment was used to manage questionnaire refinement between rounds, whereas the final inclusion of categories in the preliminary CAS was based on the Round 3 panel ratings according to the RAND/UCLA appropriateness criteria.

Items were considered for addition to a subsequent‐round questionnaire when (1) experts provided comments recommending addition or (2) the WG judged that the suggestion should be retained for panel re‐rating in the next round. Conversely, items could be removed from a subsequent‐round questionnaire when (1) the item was judged “uncertain” based on survey results, (2) the item lacked panel agreement, (3) experts provided comments recommending deletion, or (4) the WG judged that the item was redundant or insufficiently relevant for further rating.

### 2.4. Ethical Considerations

This study was conducted in accordance with the Declaration of Helsinki and received ethical approval from the Ethics Committee of the JAOT (No. E‐24001). All participants were informed in advance that participation was voluntary, that nonparticipation would incur no disadvantage, how personal data would be handled, and that completion and return of the questionnaire would constitute implied informed consent to participate.

## 3. Results

### 3.1. Delphi Process and Panel Participation

Data were collected from August 2024 to January 2025. Of the 15 experts invited, one psychiatrist declined participation after explanation of the study. The remaining 14 panelists (four psychiatrists and 10 occupational therapists) completed all the three Delphi rounds. A wide range of candidate ICF categories was proposed in Round 1, and interexpert variability was observed. Iterative feedback in Rounds 2 and 3 produced increasing convergence: the number of items judged “appropriate” in Rounds 1–3 was 50, 48, and 42, respectively. Ultimately, 42 ICF categories were retained as useful for psychiatric occupational therapy. Table 2 summarizes the consensus process across the three rounds, including the median scores and the number of ratings outside the median tertile in each round. Overall, agreement rates increased across rounds, indicating growing stability of the selected indicators. A complete item‐level summary of whether each modification was primarily panel based, WG based, or both is provided in Table S1. Across rounds, modifications to the questionnaire reflected both panel input and WG‐based refinement. Additions such as bowel function (b525), using transportation (d470), caring for body parts (d520), and basic economic transactions (d860) were prompted by panel suggestions, whereas some restructuring of environmental‐factor items was undertaken by the WG to reduce overlap and improve feasibility for the subsequent round. Final retention in the preliminary CAS was determined by the Round 3 panel ratings.

**Table 2:** Round‐by‐round progress of consensus.

	Median score (/9)^f^			Number outside median tertile (/14)^g^		
*Rounds*	*1*	*2*	*3*	*1*	*2*	*3*
b110—Consciousness functions^a^	8	—	—	**4**	—	—
b114—Orientation functions	8	8	8	2	2	1
b117**—**Intellectual functions	9	9	9	0	0	0
b122**—**Global psychosocial functions	9	8.5	8.5	2	1	1
b126**—**Temperament and personality functions	8.5	8	8	1	0	0
b130**—**Energy and drive functions	8.5	8.5	8.5	0	0	1
b134**—**Sleep functions	9	9	8.5	0	0	0
b140**—**Attention functions	9	9	9	0	0	0
b144**—**Memory functions	9	9	9	1	0	0
b147**—**Psychomotor functions	8	8	8	2	0	0
b152**—**Emotional functions	9	9	9	1	0	0
b156**—**Perceptual functions	8	8	8.5	1	1	0
b160**—**Thought functions	9	9	9	0	0	0
b164**—**Higher level cognitive functions	9	9	9	1	0	0
b167**—**Mental functions of language	7	7	8	**4**	3	3
b180**—**Experience of self and time functions	7.5	7	8	3	3	2
b420**—**Blood pressure functions^a^	**6**	—	—	**6**	—	—
b455**—**Exercise tolerance functions	7	7.5	8	3	2	1
b515**—**Digestive functions^a^	**6**	—	—	**5**	—	—
b525**—**Defecation functions^cd^	—	7	7	—	**4**	**5**
b530**—**Weight maintenance functions	7	7	7	2	2	2
b540**—**General metabolic functions^a^	**5**	—	—	**5**	—	—
b545**—**Water, mineral, and electrolyte balance functions^b^	**6.5**	7	—	**7**	**5**	—
b730**—**Muscle power functions^a^	**6**	—	—	**8**	—	—
d155**—**Acquiring skills	8	8.5	8	1	0	0
d163**—**Thinking	7.5	8	7	2	3	3
d175**—**Solving problems	9	8	8.5	0	0	0
d177**—**Making decisions	8	9	9	1	1	1
d210**—**Undertaking a single task	9	9	9	0	0	0
d220**—**Undertaking multiple tasks	9	9	8.5	1	1	1
d230**—**Carrying out daily routine	9	9	9	0	0	0
d240**—**Handling stress and other psychological demands	9	9	9	0	0	0
d350**—**Conversation	8	8	8	1	1	1
d465**—**Moving around using equipment^a^	7	—	—	**6**	—	—
d510**—**Washing oneself^a^	7	—	—	2	—	—
d470**—**Using transportation^d^	—	8	8	—	0	0
d520**—**Caring for body parts^d^	—	8	8	—	2	0
d570**—**Looking after one′s health	8	8.5	8	1	1	0
d620**—**Acquisition of goods and services	8	8	8	0	0	1
d630**—**Preparing meals	7.5	7.5	8	2	2	0
d640**—**Doing housework	8	8	8	0	0	1
d710**—**Basic interpersonal interactions	9	9	9	1	0	0
d720**—**Complex interpersonal interactions	8.5	9	8.5	0	0	0
d740**—**Formal relationships^a^	8	—	—	**4**	—	—
d760**—**Family relationships	9	8	8.5	1	1	3
d770**—**Intimate relationships^c^	7.5	7	7	**4**	3	**5**
d845**—**Acquiring, keeping, and terminating a job	8	8	8	0	1	1
d860**—**Basic economic transactions^d^	—	8	8	—	0	0
d910**—**Community life^a^	**5**	—	—	**6**	—	—
d920**—**Recreation and leisure	8	8.5	8	**4**	0	0
e1101**—**Drugs^b^	8	8	—	**4**	**4**	—
e310**—**Immediate family^b^	8	8	—	0	2	—
e320**—**Friends^a^	7.5	—	—	3	—	—
e325**—**Acquaintances, peers, colleagues, neighbors, and community members^b^	7.5	7	—	3	**4**	—
e355**—**Health professionals^a^	8	—	—	1	—	—
e410**—**Individual attitudes of immediate family members	8.5	9	9	2	**4**	1
e415**—**Individual attitudes of extended family members^c^	7	7	**6**	3	**4**	**8**
e420**—**Individual attitudes of friends^c^	7	6	7	**6**	**8**	**5**
e450**—**Individual attitudes of health professionals^c^	7.5	7.5	7	**6**	**5**	**4**
e460**—**Societal attitudes^a^	**6**	—	—	**6**	—	—
e570**—**Social security services, systems, and policies	8	8	8	0	2	0
e580**—**Health services, systems, and policies	8	8	8	0	2	0
e425**—**Individual attitudes of acquaintances, peers, colleagues, neighbors, and community members^e^	—	—	7	—	—	3
e430**—**Individual attitudes of people in positions of authority^ce^	—	—	7	—	—	**5**

*Note:* Final ICF items retained in the Core Assessment Set are indicated in **bold** in the table. Between‐round modifications reflected either panel‐based input (ratings and/or free‐text comments), WG‐based questionnaire refinement, or both. WG‐based refinement was used to reduce redundancy, improve feasibility, and reorganize overlapping categories for the subsequent round. Final inclusion in the preliminary Core Assessment Set was determined by the Round 3 panel ratings according to the RAND/UCLA appropriateness criteria. A complete item‐level classification of the basis for additions and removals across rounds is provided in Table S1.

*Note:*

^a^Removed after Round 1.

^b^Removed after Round 2.

^c^Removed after Round 3.

^d^Added after Round 1.

^e^Added after Round 2.

^f^Scores classified as “Uncertain” are shown in **bold**.

^g^Numbers of disagreement are shown in **bold**.

### 3.2. Body Functions (b Codes)

Body‐function items achieved relatively high agreement from the early rounds. Categories that consistently reached consensus included orientation (b114), attention (b140), memory (b144), and emotional (b152) functions, which are relevant across major diagnoses such as schizophrenia, depression, and bipolar disorder. Bowel function (b525) was suggested in panel comments and added in Round 2, but it was removed in Round 3 following later round review by the panel and the WG.

### 3.3. Activities and Participation (d Codes)

Activities and participation items were judged highly relevant to psychiatric occupational therapy. Items prioritized by the panel included carrying out daily routines (d230), handling stress and other psychological demands (d240), and acquiring/keeping/terminating a job (d845)—all of which relate directly to social reintegration and quality of life. Use of transport (d470), caring for body parts (d520), and basic economic transactions (d860) were proposed as additions in Round 2 based on panel suggestions and were retained in the final set. These activity/participation domains featured prominently in the final CAS.

### 3.4. Environmental Factors (e Codes)

Opinions on environmental factors were more heterogeneous. Some panelists emphasized the clinical importance of attitudes of family (e410), relatives (e415), and friends (e420), whereas others noted that such factors are difficult for occupational therapists to evaluate alone. Concerns were raised that including both family as a resource (e310) and family attitudes (e410) would substantially increase the item count. Following Round 2 discussions, the WG aimed to harmonize e3 (support and relationships) by emphasizing attitudinal subcategories of e4; consequently, acquaintance/friend/colleague/neighbor/community members (e325) was removed through panel feedback and WG review, and the attitude of acquaintances/peers (e425) was added through WG‐based questionnaire refinement. The attitude of people in positions of authority (e430) was debated and added in Round 3 through WG‐based questionnaire refinement but subsequently excluded based on Round 3 responses. Family attitudes (e410) and social security, services, systems, and policies (e570) reached consensus and were included in the final set.

### 3.5. Items Removed and Reasons for Exclusion

Most deleted items were judged to be of lower priority for psychiatric occupational therapy assessment, difficult for occupational therapists to assess independently, or effectively covered by other retained ICF categories. Some deletions were primarily driven by panel ratings or comments, whereas others reflected WG‐based refinement to reduce redundancy and improve feasibility before the next Delphi round. Examples of removed items include bowel function (b525), intimate relationships (d770), and attitudes of friends (e420).

## 4. Discussion

This Delphi study identified and reached consensus on a preliminary ICF assessment set for psychiatric occupational therapy in Japan comprising 42 categories (17 body functions, 21 activities and participation, and four environmental factors). The progressive convergence of expert opinions across three rounds supports the internal consistency of the consensus process and suitability of the Delphi method. Our work provides an initial, context‐sensitive framework for structuring occupational therapy assessment in psychiatric settings.

A key finding is that the expert panel reliably narrowed a broad pool of candidate ICF items to a focused set that emphasizes everyday functioning and participation. Items such as attention, decision‐making, and undertaking daily routines were prioritized, reflecting clinicians′ emphasis on domains that directly affect recovery and quality of life. Compared with ICF set development in other clinical areas, the iterative selection and retention of items in the present study appear methodologically consistent and clinically plausible. The prominence of activity and participation categories aligns with prior views that psychiatric rehabilitation and occupational therapy should prioritize functional engagement and social role recovery [12].

In addition, the Delphi process highlighted the tension between comprehensiveness and clinical feasibility. Given the heterogeneity of psychiatric conditions and varying individual needs, an assessment tool must balance breadth (to capture relevant problems) and parsimony (to remain usable in routine practice). Several candidate items were removed because they were judged either to be of lower priority, difficult for occupational therapists to assess independently, or subsumed by retained categories. This pruning reflects a pragmatic approach to producing a usable CAS while preserving the comprehensive conceptual scope of ICF.

The findings also echo concerns raised in the literature about the generalizability of ICF core sets across contexts. Fresk et al. (2023) reported limits to core set universality, especially in domains where diagnostic labels and functional manifestations are ambiguous. Similarly, our results indicate that standardization in psychiatric practice requires built‐in flexibility [23]. By selecting items that reflect Japan′s clinical and cultural context, the CAS aims to be both locally applicable and compatible with international frameworks, thereby facilitating cross‐national comparisons and collaborative research.

Clinically, the CAS can serve multiple roles, such as clarify assessment priorities for occupational therapists, support multidisciplinary communication through a shared language, guide intervention planning, and function as an educational scaffold for trainees. In research contexts, a standardized item set enhances reproducibility and enables aggregation of data across sites for outcome evaluation and comparative studies. Methodologically, the use of Delphi consensus to derive the CAS follows established practices in ICF core set development and is likely to be acceptable in international discourse [24].

This study has several limitations. First, the item selection process relied primarily on the perspectives of professional experts (psychiatrists and occupational therapists) and did not directly incorporate the lived‐experience perspectives of service users or their families. The inclusion of service‐user and caregiver viewpoints can ensure relevance and acceptability of assessment content and should be addressed in future work [25]. Second, this study did not evaluate the psychometric properties—reliability, validity, and responsiveness—of the selected CAS. The present results provide only content‐validity evidence via expert consensus, necessitating subsequent empirical studies to test measurement performance in clinical populations. Third, panel selection was constrained by the limited pool of professionals in Japan who are experienced in both psychiatric practice and ICF application. To mitigate this professional constraint, we used association nominations and WG oversight, but further validation with broader and more diverse stakeholder samples could strengthen generalizability.

Based on these results, the following steps are recommended: (1) empirically test the CAS in clinical samples to assess interrater reliability, construct validity (convergent/divergent), and sensitivity to change; (2) conduct stakeholder consultations—especially with service users and family members—to refine item content, wording, and acceptability; (3) develop practical scoring rules and administration guidance (including a brief clinician form and a complementary client‐reported module) to promote routine use; and (4) publish the final item list and round‐by‐round selection rates as a supplementary table to enhance transparency and enable replication.

## 5. Conclusions

This study identified ICF categories relevant to comprehensive occupational therapy assessment for people with mental disorders and produced an ICF‐based assessment set intended to support standardization of clinical evaluation. Through a three‐round Delphi process, 42 categories were retained, and the convergence of expert opinion across rounds supports the content validity and internal consistency of the selected items. The proposed assessment set offers occupational therapists a structured framework to perform comprehensive evaluations while accommodating the heterogeneity of psychiatric symptoms and functional presentations. Future work should include stakeholder consultations (service users and families), psychometric validation (reliability, validity, and responsiveness), and feasibility testing in routine clinical settings.

## Author Contributions

Conceptualization and methodology: all authors. Writing—original draft: K.K. and S.S. Project administration (Ethics submission): K.K., S.S., C.M., and T.H. Formal analysis: K.F. Data interpretation: K.F., with contributions from all authors. Writing—review and editing: T.T., T.S., T.H., and S.N.; all authors contributed to manuscript preparation and revisions.

## Funding

This study was supported by the Japanese Association of Occupational Therapists.

## Disclosure

All authors read and approved the final manuscript.

## Conflicts of Interest

The authors declare no conflicts of interest.

## Supporting information


**Supporting Information** Additional supporting information can be found online in the Supporting Information section. Table S1: Item‐level classification of the basis for additions and removals across Delphi rounds.

## Data Availability

The data that support the findings of this study are available on request from the corresponding author. The data are not publicly available due to privacy or ethical restrictions.
