# Supplementary material for: Development of an ICF‐Based Core Assessment Set for Psychiatric Occupational Therapy in Japan: A Three‐Round Delphi Study
Source: Occup Ther Int. 2026 May 4;2026:5138252. doi: 10.1155/oti/5138252 (PMC13137811; doi:10.1155/oti/5138252)
Supplement: Supplementary file 1 — Supporting Information Additional supporting information can be found online in the Supporting Information section. Table S1: Item‐level classification of the basis for additions and removals across Delphi rounds. [file OTI-2026-5138252-s001.docx]

Supplementary Table S1. Item-level classification of the basis for additions and removals across Delphi rounds

| **Round** | **ICF code** | **Modification** | **Basis of modification** |
| --- | --- | --- | --- |
| 1 | b110 | Removed | Panel-based |
| 1 | b420 | Removed | Panel-based |
| 1 | b515 | Removed | Panel-based |
| 1 | b540 | Removed | Panel-based |
| 1 | b730 | Removed | Panel-based |
| 1 | d465 | Removed | Panel + WG |
| 1 | d510 | Removed | WG-based |
| 1 | d740 | Removed | Panel + WG |
| 1 | d910 | Removed | Panel-based |
| 1 | e320 | Removed | WG-based |
| 1 | e355 | Removed | WG-based |
| 1 | e460 | Removed | Panel-based |
| 1 | b525 | Added | Panel-based |
| 1 | d470 | Added | Panel-based |
| 1 | d520 | Added | Panel-based |
| 1 | d860 | Added | Panel-based |
| 2 | b545 | Removed | Panel-based |
| 2 | e1101 | Removed | Panel-based |
| 2 | e310 | Removed | Panel + WG |
| 2 | e325 | Removed | Panel + WG |
| 2 | e425 | Added | WG-based |
| 2 | e430 | Added | WG-based |
| 3 | b525 | Removed | Panel + WG |
| 3 | d770 | Removed | Panel + WG |
| 3 | e415 | Removed | Panel-based |
| 3 | e420 | Removed | Panel-based |
| 3 | e430 | Removed | Panel-based |
| 3 | e450 | Removed | Panel-based |

Panel-based = modification primarily based on panel ratings and/or free-text comments; WG-based = modification primarily based on working group judgment during questionnaire refinement; Panel + WG = modification based on both panel input and working group review.
